# Dietary Essential Amino Acid Restriction Promotes Hyperdipsia via Hepatic FGF21

**DOI:** 10.3390/nu13051469

**Published:** 2021-04-26

**Authors:** Patricia M. Rusu, Andrea Y. Chan, Mathias Heikenwalder, Oliver J. Müller, Adam J. Rose

**Affiliations:** 1 Nutrient Metabolism & Signalling Laboratory, Department of Biochemistry and Molecular Biology, Metabolism, Diabetes and Obesity Program, Biomedicine Discovery Institute, Monash University, 3800 Clayton, Australia; andrea.chan2@monash.edu; 2 Division Chronic Inflammation and Cancer, German Cancer Research Center, 69120 Heidelberg, Germany; m.heikenwaelder@dkfz-heidelberg.de; 3 Department of Internal Medicine III, University of Kiel and DZHK (German Centre for Cardiovascular Research), 24105 Kiel, Germany; Oliver.Mueller@uksh.de

**Keywords:** amino acids, dietary protein, restriction, water intake

## Abstract

Prior studies have reported that dietary protein dilution (DPD) or amino acid dilution promotes heightened water intake (i.e., hyperdipsia) however, the exact dietary requirements and the mechanism responsible for this effect are still unknown. Here, we show that dietary amino acid (AA) restriction is sufficient and required to drive hyperdipsia during DPD. Our studies demonstrate that particularly dietary essential AA (EAA) restriction, but not non-EAA, is responsible for the hyperdipsic effect of total dietary AA restriction (DAR). Additionally, by using diets with varying amounts of individual EAA under constant total AA supply, we demonstrate that restriction of threonine (Thr) or tryptophan (Trp) is mandatory and sufficient for the effects of DAR on hyperdipsia and that liver-derived fibroblast growth factor 21 (FGF21) is required for this hyperdipsic effect. Strikingly, artificially introducing Thr de novo biosynthesis in hepatocytes reversed hyperdipsia during DAR. In summary, our results show that the DPD effects on hyperdipsia are induced by the deprivation of Thr and Trp, and in turn, via liver/hepatocyte-derived FGF21.

## 1. Introduction

Amino acids are used by the organism as the building blocks of newly synthesized proteins and as the precursors for non-proteinogenic amino acids. Therefore, protein intake and its digestion of individual amino acids are vital for life [1,2]. Interestingly, although a lack of protein can have severe effects and is not compatible with life, recent studies have shown that reduced protein intake to a certain threshold, can actually lead to significant health benefits [3,4,5]. Several pre-clinical investigations have shown that dietary protein restriction (DPR) has positive effects on several age-related pathologies such as Alzheimer’s disease, type 2 diabetes, and likely also certain cancers [6,7,8,9,10,11,12]. It is important to note that the responses to DPR are conserved from mice to humans as shown in several human trials [8,11,13]. In addition, studies of human populations have shown that type 2 diabetes risk [14] and all-cause mortality [15] positively correlate with protein intake, especially that from animal sources.

Many of the effects of DPD on systemic metabolism can be ascribed to increased liver-derived secretion and action of the peptide hormone FGF21 [5,16], including effects on metabolic inefficiency [8,17,18,19,20] as well as glucose [7,8,17] and lipid [9,21], but not protein [8], homeostasis. In addition, several recent reports have indicated that FGF21 per se can stimulate water intake [22,23] and the water intake of mice is also higher when undergoing dietary amino acid restriction [24]. The biological basis for FGF21-stimulated water intake and the precise nutritional components driving increased water intake during DPD are not presently clear. Thus, in this study, we examined whether dietary protein restriction could stimulate water intake, as well as the nutritional components and hormonal mechanisms behind this response.

## 2. Materials and Methods

### 2.1. Diets

**Figure 1:**
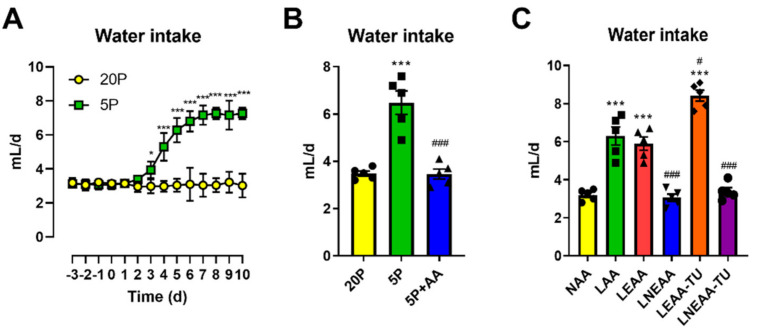
Dietary restriction of essential-, but not non-essential-, -amino acids is sufficient and required for the hyperdipsic response to dietary protein dilution. (**A**): Time-course effect of dietary protein dilution on water intake. Both groups were fed a control diet (20P) leading up to the diet switch, where one group was switched (time 0) to a protein dilute diet (5P) with water intake measured throughout. Data are mean and SEM; *n* = 8 per group. Different than 20P: * *p* < 0.05, *** *p* < 0.001. (**B**): Water intake rate of mice kept on diets with 20% energy from protein (20P), 5% energy from protein (5P), or 5% energy from protein and 15% energy from amino acids (AA) to match 20P. Data are mean ± SEM; *n* = 5 per group. Different than 20P: *** *p* < 0.001. Different than 5P: ^###^ *p* < 0.001. (**C**): Water intake in mice fed diets containing 18% AA (normal AA; NAA), 4.5% AA (low AA; LAA), low essential AA (EAA) with normal non-EAA (LEAA), low non-EAA with normal EAA (LNEAA), LEAA with NEAA topped up to reach total AA of that in NAA (LEAA-TU), and LNEAA with EAA topped up to reach total AA of that in NAA (LNEAA-TU). Data are mean ± SEM; *n* = 5 per group. Different than 20P: *** *p* < 0.001. Different than 5P: ^#^ *p* < 0.05, ^###^ *p* < 0.001. Statistical analyses: Two-way repeated measures ANOVA (**A**), one-way ANOVA (**B**,**C**).

**Figure 2:**
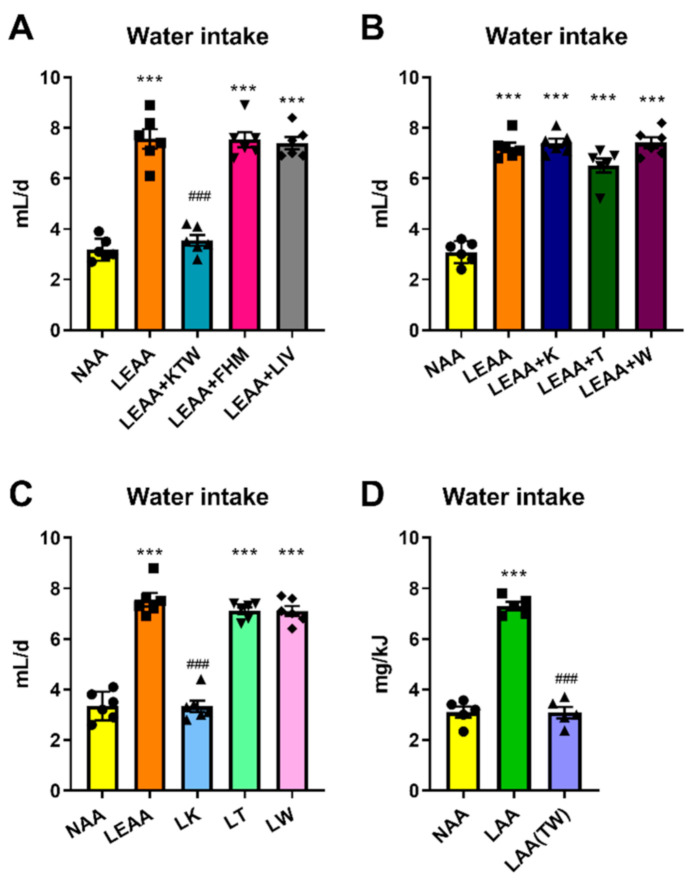
Restriction of Threonine and Tryptophan is sufficient and necessary for the effect of dietary protein dilution on hyperdipsia. (**A**): Water intake rate of mice fed a normal amino acid diet (NAA, 18% calories from AA), a low essential amino acid diet (LEAA) with EAA at values of a 4.5% total AA diet, and a LEAA diet supplemented with either lysine, threonine, and tryptophan (LEAA + KTW), phenylalanine, histidine, and methionine (LEAA + FHM), or leucine, isoleucine, and valine (LEAA + LIV). All diets were adjusted to 18% AA in total by non-essential AA (NEAA) top-up. Data are mean ± SEM (*n* = 6 per group). Different than diet NAA: *** *p* < 0.001. Different than diet LEAA: ^###^ *p* < 0.001. (**B**): Water intake of mice treated with NAA, LEAA, and LEAA supplemented with either lysine (LEAA + K), threonine (LEAA + T), and tryptophan (LEAA + W), all with NEAA equally adjusted to give 18%AA in total. Data are mean ± SEM; *n* = 6 mice per group. Different than diet NAA: *** *p* < 0.001. (**C**): Water intake of mice in response to treatment with the following diets NAA, LEAA, and diets with restricted amounts of either lysine (low lysine, LK), threonine (LT), and tryptophan (LW). All diets were equally adjusted with the other AA to give 18%AA in total. Data are mean ± SEM; *n* = 6 per group. Different than diet NAA: *** *p* < 0.001. Different than diet LEAA: ^###^ *p* < 0.001. D: Water intake of mice in response to treatment with the following diets: NAA (normal amino acid, 18% energy from AA), LAA (low AA, 4.5% AA as used in Figure 1C), and LAA supplemented with threonine and tryptophan while keeping total AA at 4.5% (LAA(TW). Data are mean ± SEM (*n* = 5 per group). Different than NAA: *** *p* < 0.001. Different than LAA: ^###^ *p* < 0.001. Statistical analyses: One-way ANOVA (**A**–**D**).

**Figure 3:**
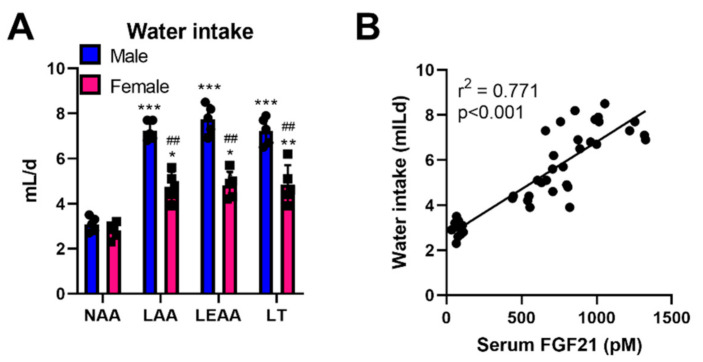
The hyperdipsic response to dietary AA restriction also occurs in fully mature female and male mice. Water intake of 6-month-old female and male mice treated with a normal amino acid diet (NAA, 18% energy from amino acids), a low AA diet (LAA, 4.5%), a low essential amino acid diet (LEAA) with EAA like in LAA and with non-EAA top-up to 18%, and a diet low in Threonine but with matching total AA to NAA (LT). Data are mean ± SEM (*n* = 5 per group). Data were analysed by two-way ANOVA. Different than diet NAA: * *p* < 0.05, ** *p* < 0.01, *** *p* < 0.001. Different than diet male: ^##^ *p* < 0.01. (**B**): Scatter plot of water intake and serum FGF21 levels of mice as in (**A**). Shown are r^2^ and *p* values from Pearson’s correlation.

**Figure 4:**
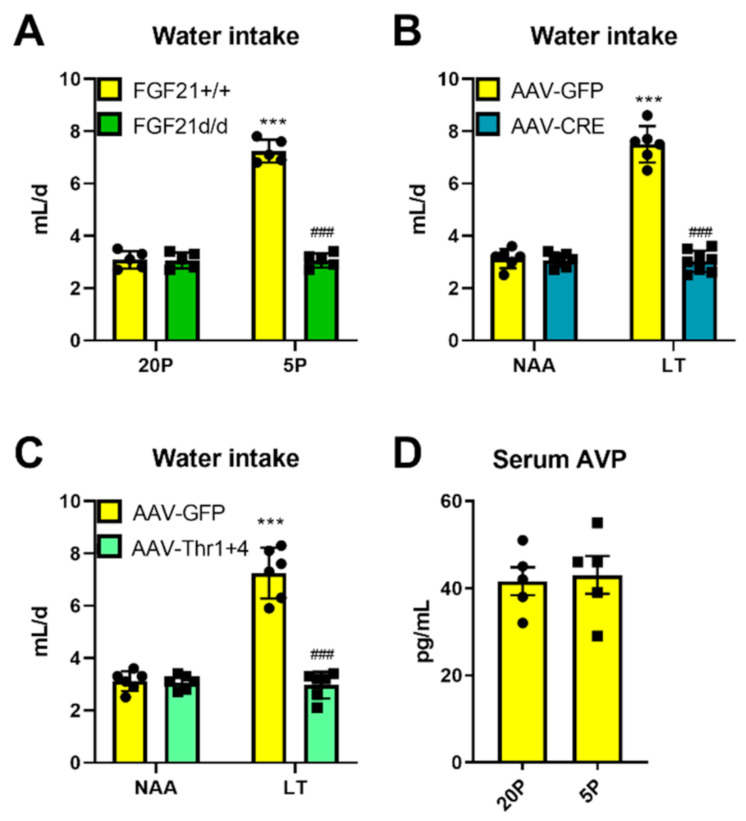
Liver-derived FGF21 is required for the hyperdipsic response to dietary AA restriction. (**A**): Water intake rate of wildtype (Fgf21+/+) and whole body knockout (Fgf21d/d) littermate mice in response to treatment with diets containing 20% calories from protein (20P) or 5% calories from protein (5P). Data are mean ± SEM (*n* = 5 per group). Different than 20P: *** *p* < 0.001. Different than Fgf21+/+: ^###^ *p* < 0.001. (**B**): Water intake of Fgf21fl/fl mice treated with a normal amino acid diet (NAA, 18% energy from amino acids) or with a diet low in Threonine but with matching total AA to diet NAA (LT). Mice were pre-treated (tail vein injection) with adeno-associated viruses to express the Cre-recombinase (AAV-CRE) or the green fluorescent protein (AAV-GFP) in a hepatocyte-selective manner. Data are mean ± SEM (*n* = 6–8 per group). Different than NAA: *** *p* < 0.001. Different than AAV-GFP: ^###^ *p* < 0.001. (**C**): Water intake of mice kept on NAA or LT diet following tail vein injection of adeno-associated viruses to transduce the liver to either express the yeast threonine biosynthetic enzymes Thr1 and Thr4 or GFP as a negative control. Data are mean ± SEM (*n* = 6 per group). Different than diet NAA: *** *p* < 0.001. Different than AAV-GFP: ^###^ *p* < 0.001. (**D**): Serum arginine-vasopressin levels of mice in response to treatment with diets containing 20% energy from protein (20P) or 5% energy from protein (5P). Data are mean ± SEM; *n* = 5 per group. Statistical analyses: Two-way measures ANOVA (**A**–**C**), student’s *t*-test (**D**).

**Figure 5:**
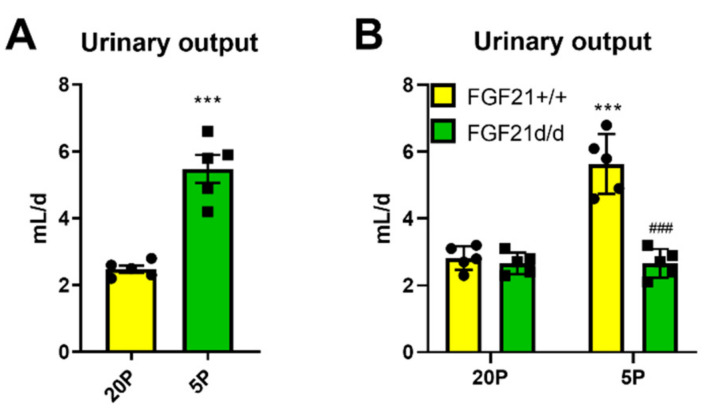
Liver-derived FGF21 is necessary for the heightened urinary response to dietary protein restriction. (**A**): Water intake rate of mice in response to treatment with diets containing 20% energy from protein (20P) or 5% energy from protein (5P). Data are mean ± SEM; *n* = 5 per group. Different than 20P. (**B**): Urinary volume output rate of wildtype (Fgf21+/+) and germline knockout (Fgf21d/d) littermate mice in response to treatment with diets containing 20% calories from protein (20P) or 5% calories from protein (5P). Data are mean ± SEM (*n* = 5 per group). Different than 20P: *** *p* < 0.001. Different than Fgf21+/+: ^###^ *p* < 0.001. Statistical analyses: Student’s *t*-test (**A**), two-way measures ANOVA (**B**).

Semi-pure pelleted diet stocks were purchased from either from Specialty Feeds (Perth, Australia; Supplementary Table S1) or Research Diets (New Brunswick, NJ, USA; Supplementary Table S2). Experiments of Figures 1A, 4A,D and 5A,B used SF17-180 and SF17-175. Experiments of Figure 1B used SF17-180, SF17-175, and SF17-176. Experiments of Figure 1C) used A14011601-A14011606. Experiments of Figure 2A used A14011601, A14011605, and A16120501-A16120503. Figure 2B used A14011601, A14011605, and A17020901-A17020903. Experiments of Figure 2C used A14011601, A14011605, and A170401301-A170401303. Experiments of Figure 2D used SF17-177, SF18-109, and SF17-110. Experiments of Figure 3A,B used SF17-177, SF18-109, SF19-086, and SF17-179. Figure 4B used SF17-177 and SF17-179. Figure 4C used SF17-177 and SF18-066.

### 2.2. Recombinant Viruses

Yeast (*Saccharomyces cerevisiae*) Threonine 1 (Thr1; homoserine kinase), or Threonine 4 (Thr4; threonine synthase), a control (green fluorescent protein: GFP), and Cre-recombinase were individually cloned into an adeno-associated virus (AAV) genome plasmid behind the LP1 promoter, using standard techniques. Importantly, the LP1 promoter was only active in hepatocytes [25,26,27]. In turn, self-complimentary AAVs were produced by triple transfecting HEK293T cells with the individual genome plasmids, the helper plasmid pDG∆VP, and the capsid plasmid p5E18VD2/8-mut6 (AA 589-592: QNTA to GNRQ) [28]. This was followed by density gradient/filtration purification and titration by quantitative real-time PCR [29].

### 2.3. Mouse Experiments

Mice (7 weeks) were acclimated to the local experimentation facility 12–12 h light-dark cycle, 22–24 °C) on arrival for 1 week. Mice were of the C57Bl/6J strain (Monash University Animal Research Platform, Clayton, AUS or Animal Resource Centre, AUS). Fgf21-fl/fl and global germline knockout littermate mice [30] were also used. The dietary intervention was identical for nearly all experiments (Figures 1B,C, 2A–D and 4A–C). In brief, following acclimation, mice were placed on diets for 3 weeks with body weight recorded each week with metabolic cage Promethion-M High Definition Multiplexed Respirometry System (Sable Systems International, Las Vegas, NV, USA)) housing for 5 days during week 2. Water intake was measured by individual housing in metabolic phenotyping system cages, and in some studies, urinary volume output was measured in custom metabolic cages for urine collection [8], directly after metabolic cage housing. Some findings from these experiments were previously published [31].

In addition to the studies on young mice, 6 mo old male and female C57Bl/6J mice were fed diets SF17-177 (NAA), SF18-109 (LAA), SF19-086 (LEAA), and SF17-179 (LT) for 8 weeks. Promethion system individual cage experiments were conducted for 5 days, 7 days after initial diet feeding.

For hepatocyte Fgf21 knockout experiments (Figure 4B) Fgf21fl/fl mice (7 week male) were intravenously administered 2.5 × 10^11^ viral genomes per mouse of either control (GFP) or Cre-recombinase (CRE) expressing; via the tail vein. One week after diet feeding was initiated and continued for 8 weeks, and mice were then euthanized for tissue collection. During week 2, individual mouse Promethion cage measurements were made.

For liver/hepatocyte-specific yTHR1 and yTHR4 expression experiments (Figure 4C), mice were administered a total of 5 × 10^11^ viral genomes via the tail vein. The dietary intervention was started one week after AAV administration.

### 2.4. Blood Serum Hormone Analyses

Blood serum FGF21 (MF2100, R&D Systems) and arginine-vasopressin (AVP, also known as anti-diuretic hormone; LS-F7592, LSBio) were measured from the blood serum using commercially available kits. Importantly, kits were used following the instructions from the manufacturer, and values recorded always fell within the standard curve.

### 2.5. Statistical Analysis

One- or two-way analysis of variance (ANOVA), with or without repeated measures, were conducted for most experiments, with Holm–Sidak-adjusted post-tests. Statistical analyses were conducted using SigmaPlot 14 (Systat Software, Inc. San Jose, CA, USA), and data were visualized using GraphPad Prism 8.0 (GraphPad Software, Inc., San Diego, CA, USA). Differences were noted as significant with a *p* value less than 0.05.

## 3. Results

### 3.1. Dietary Protein Dilution Increases Water Intake via Dietary Essential Amino Acid Restriction

To begin with, we examined the effects of a dilution of dietary protein (DPD) on water intake over time (Figure 1A). Importantly, the ~3 mL/d water intake on the control diet was within the typical range of laboratory mice [32]. Of note, DPD caused a significant increase after 3 days (~25%) with a sustained increase (~100%) in water intake (i.e., hyperdipsia) after 6–7 days (Figure 1A). As several of the effects of DPD were due to amino acid restriction [32], we then examined whether this could also be true for altered water intake. Indeed, when we topped up a protein diluted diet with amino acids (5P + AA) to match that of the control diet (20P), the higher water intake with DPD was completely blunted (Figure 1B). However, since the total carbohydrate content of these diets was simultaneously altered to keep them isocaloric, this study could not discern whether the difference was specifically due to altered protein/AA or carbohydrate. To address this, as well as which particular class of AA (essential or non-essential [2]) were required for the effects of DPD on hyperdipsia, we then performed an experiment where EAA and NEAA were specifically manipulated (Figure 1C). Whether an AA was EAA or NEAA was defined on the basis of nutrition studies showing a failure to thrive when a single AA was absent in the diet fed to adult rodents under standard laboratory conditions [1,2]. Additionally, we used diets which were topped up with the alternate source of AA to keep the total AA supply constant without altering dietary fat or carbohydrate supply (Figure 1C). As shown in Figure 1C, a selective restriction of EAA fully reproduced the effects of DPD on heightened water intake, with an even higher effect when EAA restriction was combined with NEAA top-up to keep the nitrogenous calories equal to the control diet group (NAA).

### 3.2. DPD Effects on Water Intake Are Dependent on Thr and Trp Restriction

We then investigated which particular EAA could confer the effect of dietary AA restriction on increased water intake. For this purpose, we divided the nine EAAs into three subgroups based on their biochemical characteristics [2]. The first group contained the EAAs, which cannot be synthesized from any metabolite in the mammalian metabolic biochemical network (i.e., Lys, Thr, Trp). The second group included the branched-chain AAs (i.e., Ile, Leu, Val), while the third group included the three remaining EAAs (i.e., His, Met, Phe). In the following studies we supplemented the low EAA diet with these three groups of EAAs and could show that only the strictly metabolically essential AAs, namely Lys, Thr, and Trp, were required to confer the hyperdipsic response to total EAA restriction (Figure 2A). Adding back any of these strictly EAAs individually did not abolish the hyperdipsic effect of dietary EAA restriction (Figure 2B). Therefore, the restriction of at least two, if not all three, of these EAAs was required for the full effects of dietary EAA restriction on water intake. To examine this further, we carried out two additional studies. In the first study, we selectively restricted either Lys, Thr, or Trp and showed that depletion of either Trp or Thr was enough to induce the hyperdipsic effect of dietary EAA restriction (Figure 2C). In the second study, we supplemented both Trp and Thr while maintaining a low total AA supply, and could demonstrate that restoring Trp and Thr levels counteracts the hyperdipsic effect of dietary EAA restriction (Figure 2D). In conclusion, we could demonstrate that individual restriction of either Thr or Trp can induce hyperdipsia and that the addition of both of these EAAs is necessary to abolish hyperdipsia in mice fed a protein or AA restricted diet.

### 3.3. Hyperdipsia during Dietary AA Restriction also Occurs in Fully Mature Female and Male Mice

The studies presented in Figure 1 and Figure 2 were performed on 8 weeks old male mice, which were relatively young and still growing and thus might have different AA requirements than adult mice. Additionally, it has been shown that female mice respond differently to dietary challenges [33,34,35]. To prove that hyperdipsia is a general response to low dietary AA, we tested the previously used diets (Figure 1 and Figure 2) on 6 months old male and female mice to assess potential differences (Figure 3). In all forms of dietary AA restriction, water intake rates were higher, with a lower response in female mice (Figure 3A). Given that we also recorded lower FGF21 responses in female mice during dietary AA restriction, we assessed the potential relationship between these variables. In particular, the water intake rates were positively correlated to FGF21 levels (Figure 3B).

### 3.4. Liver-FGF21 Is Required for Dietary AA Restriction Effects on Water Intake

Given the positive correlation between FGF21 and water intake with dietary AA restriction, we then tested whether FGF21 was required for the heightened water intake with dietary AA restriction. Firstly, we tested this in whole body FGF21 knockout mice, which did not show any increase of FGF21 in blood serum in response to DPD [8]. While water intake was higher with dietary protein restriction in mice with FGF21, littermate mice without FGF21 lacked this response (Figure 4A). In addition, adult mice with hepatocyte-selective genetic silencing of Fgf21 also lacked the hyperdipsic response to a low Thr diet (Figure 4B). The metabolic turnover of each EAA was related to its requirement, which is encoded in the exome [36], however, EAAs can have other dominant metabolic roles other than protein synthesis, such as neurotransmitter synthesis [2]. Therefore, we decided to test whether it was the inability to synthesize the strictly metabolic EAA Thr that characterizes it as limiting and thus necessary for adaptation to a restriction of EAA in the diet. As yeast can synthesize Thr, we manufactured adeno-associated viruses that expressed the yeast Thr biosynthetic enzymes (i.e., Thr1 and Thr4) behind a liver-specific promoter and used them to give the liver capacity to synthesize Thr in mice subjected to a low Thr diet. Our former studies showed that the THR1/4 expression was achieved and that this reversed the lower liver threonine levels during low threonine feeding back to that of the control diet [31]. Interestingly, by artificially inducing Thr de novo biosynthesis in hepatocytes, the effects of a low Thr diet on hyperdipsia were entirely reversed (Figure 4C). Importantly, vasopression, a key regulator of fluid osmolality/balance and thus thirst [37], was not altered by DPR (Figure 4D).

### 3.5. Urinary Volume Output Is Higher in Mice during Dietary Protein Restriction, and this Is Mediated by FGF21

Given the large effects on drinking behavior, we also examined urinary volume output. In particular, urinary volume output was doubled in DPD mice (Figure 5A). This increase in urinary volume output was due to FGF21, as FGF21 knockout mice did not have higher urinary output when subjected to DPD (Figure 5B).

## 4. Discussion

Here we demonstrate that heightened water intake during dietary protein dilution is due to the restriction of AA per se (Figure 1, Figure 2, Figure 3 and Figure 4). As an exemplar, replenishing the limiting EAA Trp and Thr while still feeding a low AA diet completely blunted the heightened water intake (Figure 2D). Furthermore, genetically enforced hepatic Thr biosynthesis reduced water intake in response to dietary Thr restriction (Figure 4C and [31]). In addition, all our studies of protein/AA restriction that showed hyperdipsia (Figure 1, Figure 2 and Figure 3) also showed higher serum levels of FGF21 [31], and our studies using genetic silencing show that the hyperdipsic response is fully dependent on the liver/hepatocyte derived peptide hormone FGF21 (Figure 4A,B). Importantly, our studies are consistent with prior observations showing that a low AA diet [24] or a low protein-ketogenic diet [22] can induce water intake, via the hormone FGF21 [22].

From our studies we could show that both simultaneous restriction of Trp and Thr were necessary for dietary AA restriction (Figure 2A,D) to induce hyperdipsia, but individual restriction of either Trp or Thr was adequate to induce hyperdipsia (Figure 2C). This is because when all EAA was restricted, and we singly add back either Thr or Trp, it did not reverse the increase in FGF21-driven hyperdipsia, because either Thr or Trp was still restricted and thus sufficient to induce hyperdipisia via FGF21 (Figure 4). In addition, others have demonstrated that restriction of sulfur-containing amino acids (SCAA) was sufficient to induce hyperdipsia in mice [38,39]. In the present studies, we did not reveal that these amino acids were critical for the induction of hyperdipsia (Figure 1). While the discrepancies between these studies were not presently clear, it could be related to that both sulfur-containing amino acids methionine and cysteine were never simultaneously restricted and perhaps did not reach a certain threshold restriction level [39]. Indeed, others have shown that restriction of both SCAA is required for physiological effects [40]. This is perhaps due to metabolic compensation, which is supported by our studies on liver threonine metabolism (Figure 4C) and [31], where ectopic expression of threonine biosynthesis enzymes reversed the effects of dietary threonine restriction.

The physiological reason why a mammal would increase water intake in response to dietary AA restriction is not presently clear. Whatever the case, this is unlikely due to an increased production of the major urinary component urea, as ureagenesis is lower with dietary protein restriction [8] and results from our AA diet studies have disconnected ureagenesis from FGF21 and water intake (Figure 2 and [31]). A previous study demonstrated that FGF21 signals to the brain to directly increase fluid ingestive behaviour [22]. On the other hand, others have shown that the increase in fluid intake is a result of the effect of FGF21 on blood pressure, which subsequently drives excessive renal fluid output and consequent hyperdipsia [23]. Indeed, we could also show that urinary volume output was higher with dietary protein dilution (Figure 5). However, that FGF21-driven blood pressure drives increased urination/drinking is unlikely to be the case with dietary protein restriction, which typically results in lower blood pressure [12]. Nevertheless, both of these studies suggested that FGF21′s effects are driven by an increased sympathetic efferent tone [22,23], which is congruent with other studies showing that many physiological effects of FGF21 depend on sympathetic nervous system activity [41,42], and that metabolite uptake is enhanced in sympathetically innervated tissues such as brown adipose and heart [8,9]. Intriguingly, others have hypothesized that the increased water intake rates are somehow linked to increased energy expenditure via FGF21 during DPD [22]. It will be interesting to further determine the mechanisms and the physiological basis behind hyperdipsia upon dietary AA restriction.

In summary, dietary essential amino acid restriction induces hyperdipsia via liver-derived FGF21.

## Data Availability

The data that form the basis of the figures presented can be provided by the corresponding authors on reasonable request.

## Supplementary Materials

The following are available online at https://www.mdpi.com/article/10.3390/nu13051469/s1, Table S1: Diet formulations from Specialty Feeds; Table S2: Diet formulations from Research Diets.
