# Supplementary material for: Dietary Essential Amino Acid Restriction Promotes Hyperdipsia via Hepatic FGF21
Source: Nutrients. 2021 Apr 26;13(5):1469. doi: 10.3390/nu13051469 (PMC8144947; doi:10.3390/nu13051469)
Supplement: Supplementary file 1 [file nutrients-13-01469-s001.zip › nutrients-1143342-supplementary.pdf]

Splementary Table 1. Diet formulations from Specialty Feeds.

Ingredient	Diet Number SF17-180 g/Kg	SF17-175	SF17-176	SF17-177	SF17-179	SF18-066	SF18-109	SF18-110	SF14-162	SF17-144	SF17-115	SF19-086
Sucrose	333.14	237.92	337.78	340.17	339.93	340.11	340.17	340.17	100	100	100	340.12
Casein	190.37	47.58	48.25						200	34.88	159.5	
L Methionine	2.86	0.71	3.65	4.9	5.05	4.9	1.22	1.03	3	0.78	12.48	1.22
L Alanine			3.65	4.9	5.05	4.9	1.22	1.03		3.47		7.52
L Arginine			4.28	5.74	5.92	5.74	1.44	1.22		4.51		8.82
L Asparagine			5.03	6.75	6.96	6.75	1.69	1.43		3.74		10.38
L Aspartic Acid			3.65	4.9	5.05	4.9	1.22	1.03		4		7.52
L Cystine			1.61	1.19	1.22	1.19	0.3	0.25		0.24		1.82
L Glutamic Acid			14.97	20.1	20.73	20.1	5.02	4.26		13.4		30.86
L Glutamine			12.32	16.55	17.07	16.55	4.14	3.51		11.27		25.42
Glycine			2.13	2.87	2.95	2.87	0.72	0.61		2.22		4.41
L Histidine			3.27	4.39	4.53	4.39	1.1	0.93		3.22		1.1
L Isoleucine			5.4	7.26	7.49	7.26	1.82	1.54		7.83	1.89	1.82
L Leucine			11.32	15.2	15.68	15.2	3.8	3.22		14.26	3.48	3.8
L Lysine			9.44	12.67	13.07	12.67	3.17	2.69		8.96		3.17
L Phenylalanine			6.04	8.11	8.36	8.1	2.03	1.72		5.83		2.03
L Proline			12.7	17.06	17.59	17.05	4.27	3.62		12.31		26.2
L Serine			7.17	9.63	9.94	9.63	2.41	2.04		6.62		14.79
L Threonine			5.15	6.92	1.73	1.73	1.73	6.29		0.23	9.61	1.73
L Tryptophan			1.51	2.02	2.09	2.02	0.51	2.02			2.03	0.51
L Tyrosine			6.54	8.79	9.06	8.78	2.2	1.87		6.24		13.49
L Valine			6.66	8.94	9.94	8.94	2.24	1.9		10.44	2.54	2.24
L Homoserine						5.35						
Soybean Oil	23.8	23.79	24.13	24.3	24.28	24.29	24.29	24.29	70	70	70	24.29
Lard	19.04	19.03	19.3	19.44	19.42	19.43	19.44	19.44				19.44
Cellulose	47.59	47.58	48.25	48.59	48.56	48.59	48.59	48.59	50	90.65	63.24	48.59
Wheat Starch	299.83	501.78	304	306.15	305.95	306.11	423.07	423.15	404.41	403.19	403.91	306.11
Dextrinised Starch	33.31	71.38	33.78	34.02	33.99	34.01	43.74	43.73	132	132	132	34.01
AIN93 Trace Minerals	1.33	1.33	1.35	1.36	1.36	1.36	1.36	1.36	1.4	1.4	1.4	1.36
Calcium Carbonate	5.24	5.23	5.31	5.35	5.34	5.34	5.35	5.34	13.13	13.1	13.13	5.34
Salt	2.47	2.47	2.51	2.53	2.53	2.53	2.53	2.53	2.59	2.59	2.59	2.53
Potassium Dihydrogen Phosphate									6.86	10.11	6.04	
Sodium Bicrbonate			7.24	7.29	7.28	7.29	7.29	7.29				7.29
Potassium Sulphate	1.52	1.52	1.54	1.56	1.55	1.55	1.56	1.56	1.63	7.53	1.18	1.55
Potassium Citrate	15.71	15.71	15.92	16.04	16.03	16.03	16.04	16.03	2.48	2.48	2.48	16.03
Dicalcium Phosphate	12.37	12.37	12.55	12.63	12.63	12.63	12.63	12.63				12.63
AIN 93 Vitamins	9.52	9.52	9.65	9.72	9.71	9.72	9.72	9.72	10	10	10	9.72
Choline Chloride 75%	1.9	1.91	1.93	1.94	1.94	1.94	1.94	1.94	2.5	2.5	2.5	1.94
Red Food Colour (124)		0.02	0.02									
Blue Food Colour 10% (133)		0.15										0.19
Egg Yellow Food Colour (102)				0.02								
Green Food Colour (133, 102)					0.02			0.02				
Sunset Yellow Food Colour (110)						0.05	0.03					
Total	1000	1000	1000	1000	1000	1000	1000	1000	1000	1000	1000	1000

Supplementary Table 2. Diet formulations from Research Diets.

Product #	A14011601		A14011602		A14011603		A14011604		A14011605		A14011606	
	100% AA		25% AA		25% EAA, 100% NE		100% EAA, 25% NE		25% EAA, 154% NE		205% EAA, 25% NE	
	41.7% EAA, 58.3% NE		41.7% EAA, 58.3% NE		15.2% EAA, 84.8% NE		74.1% EAA, 25.9% NE		10.4% EAA, 89.6% NE		85.4% EAA, 14.6% NE	
	gm	kcal	gm	kcal	gm	kcal	gm	kcal	gm	kcal	gm	kcal
Protein	16.8	17.6	4.2	4.4	11.6	12.1	9.5	9.9	16.8	17.6	16.8	17.6
Carbohydrate	68.7	72.1	81.3	86.3	74.0	77.6	76.1	79.8	68.7	72.1	68.7	72.1
Fat	4.4	10.3	4.4	10.3	4.4	10.3	4.4	10.3	4.4	10.3	4.4	10.3
Total		100.0		100.0		100.0		100.0		100.0		100.0
kcal/gm	3.8		3.8		3.8		3.8		3.8		3.8	
Ingredient (gm)												
L-Histidine-HCl-H2O	4.52	18.08	1.13	4.5	1.13	4.5	4.52	18.08	1.13	4.5	9.25	37.0
L-Isoleucine	7.47	29.88	1.87	7.5	1.87	7.5	7.47	29.88	1.87	7.5	15.31	61.2
L-Leucine	15.64	62.56	3.91	15.6	3.91	15.6	15.64	62.56	3.91	15.6	32.03	128.1
L-Lysine-HCl	13.04	52.16	3.26	13.0	3.26	13.0	13.04	52.16	3.26	13.0	26.70	106.8
L-Methionine	5.04	20.16	1.26	5.0	1.26	5.0	5.04	20.16	1.26	5.0	10.32	41.3
L-Phenylalanine	8.34	33.36	2.09	8.4	2.09	8.4	8.34	33.36	2.09	8.4	17.08	68.3
L-Threonine	7.12	28.48	1.78	7.1	1.78	7.1	7.12	28.48	1.78	7.1	14.59	58.4
L-Tryptophan	2.08	8.32	0.62	2.1	0.62	2.1	2.08	8.32	0.62	2.1	4.27	17.1
L-Valine	9.20	36.8	2.30	9.2	2.30	9.2	9.20	36.8	2.30	9.2	18.86	75.4
L-Alanine	5.04	20.16	1.26	5.0	5.04	20.2	1.26	5.04	7.74	31.0	1.26	5.0
L-Arginine	5.91	23.64	1.48	5.9	5.91	23.6	1.48	5.92	9.08	36.3	1.48	5.9
L-Asparagine-H2O	6.95	27.8	1.74	7.0	6.95	27.8	1.74	6.96	10.68	42.7	1.74	7.0
L-Aspartate	5.04	20.16	1.26	5.0	5.04	20.2	1.26	5.04	7.74	31.0	1.26	5.0
L-Cystine	1.22	4.88	0.31	1.2	1.22	4.9	0.31	1.24	1.87	7.5	0.31	1.2
L-Glutamine	17.03	68.12	4.26	17.0	17.03	68.1	4.26	17.04	26.16	104.6	4.26	17.0
L-Glutamic Acid	20.68	82.72	5.17	20.7	20.68	82.7	5.17	20.68	31.76	127.0	5.17	20.7
Glycine	2.95	11.8	0.74	3.0	2.95	11.8	0.74	2.96	4.54	18.2	0.74	3.0
L-Proline	17.55	70.2	4.39	17.6	17.55	70.2	4.39	17.56	26.96	107.8	4.39	17.6
L-Serine	9.91	39.64	2.48	9.9	9.91	39.6	2.48	9.92	15.22	60.9	2.48	9.9
L-Tyrosine	9.04	36.16	2.26	9.0	9.04	36.2	2.26	9.04	13.88	55.5	2.26	9.0
Corn Starch	315	1260	435.2	1741	359.2	1437	381	1524	315	1260	315	1260
Maltodextrin 10	35	140	45	180	45	180	45	180	35	140	35	140
Sucrose	350	1400	350	1400	350	1400	350	1400	350	1400	350	1400
Cellulose	50	0	50	0	50	0	50	0	50	0	50	0
Soybean Oil	25	225	25	225	25	225	25	225	25	225	25	225
Lard	20	180	20	180	20	180	20	180	20	180	20	180
Sodium Bicarbonate	7.5	0	7.5	0	7.5	0	7.5	0	7.5	0	7.5	0
S10026	10	0	10	0	10	0	10	0	10	0	10	0
DiCalcium Phosphate	13	0	13	0	13	0	13	0	13	0	13	0
Calcium Carbonate	5.5	0	5.5	0	5.5	0	5.5	0	5.5	0	5.5	0
Potassium Citrate, 1 H2O	16.5	0	16.5	0	16.5	0	16.5	0	16.5	0	16.5	0
Vitamin Mix V10001	10	40	10	40	10	40	10	40	10	40	10	40
Choline Bitartrate	2	0	2	0	2	0	2	0	2	0	2	0
FD&C Yellow Dye #5	0.05	0	0.025	0	0	0	0.025	0	0	0	0	0
FD&C Red Dye #40	0	0	0.025	0	0.05	0	0	0	0	0	0.025	0
FD&C Blue Dye #1	0	0	0	0	0	0	0.025	0	0.05	0	0.025	0
Total	1033.32	3940	1033.22	3940	1033.19	3940	######	3940	1033.30	3940	1033.31	3940
Percent EAA (w/w)	41.7		41.7		15.2		74.1		10.4		85.4	
Percent NEAA (w/w)	58.3		58.3		84.8		25.9		89.6		14.6	

Product #	A18120601		A18120602		A18120603		A17020901		A17020902		A17020903	
							100% Lys		100% Thr		100% Trp	
	19.3% EAA, 80.7% NE		17.5% EAA, 82.5% NE		23.5% EAA, 76.5% NE		15.5% EAA, 84.5% NE		13.2% EAA, 86.8% NE		11.2% EAA, 88.8% NE	
	gm	kcal	gm	kcal	gm	kcal	gm	kcal	gm	kcal	gm	kcal
Protein	16.8	17.6	16.8	17.6	16.8	17.6	16.9	17.7	16.8	17.7	16.8	17.6
Carbohydrate	68.7	72.1	68.7	72.1	68.7	72.1	68.7	72.0	68.7	72.1	68.7	72.1
Fat	4.4	10.3	4.4	10.3	4.4	10.3	4.4	10.3	4.4	10.3	4.4	10.3
Total		100.0		100.0		100.0		100.0		100.0		100.0
kcal/gm	3.8		3.8		3.8		3.8		3.8		3.8	
Ingredient (gm)												
L-Histidine-HCl-H2O	1.02	4.1	4.62	18.1	0.96	3.8	1.07	4.3	1.10	4.4	1.12	4.5
L-Isoleucine	1.68	6.7	1.72	6.9	7.47	29.9	1.77	7.1	1.81	7.2	1.85	7.4
L-Leucine	3.52	14.1	3.60	14.4	15.64	62.6	3.70	14.8	3.79	15.2	3.88	15.5
L-Lysine-HCl	13.04	52.2	3.00	12.0	2.78	11.1	13.04	52.2	3.16	12.6	3.23	12.9
L-Methionine	1.14	4.6	6.04	20.2	1.08	4.3	1.19	4.8	1.22	4.9	1.25	5.0
L-Phenylalanine	1.88	7.5	8.34	33.4	1.78	7.1	1.98	7.9	2.03	8.1	2.07	8.3
L-Threonine	7.12	28.5	1.64	6.6	1.52	6.1	1.69	6.8	7.12	28.5	1.76	7.0
L-Tryptophan	2.08	8.3	0.48	1.9	0.44	1.8	0.49	2.0	0.50	2.0	2.08	8.3
L-Valine	2.07	8.3	2.12	8.5	9.20	36.8	2.18	8.7	2.23	8.9	2.28	9.1
L-Alanine	6.97	27.9	7.13	28.5	6.61	26.4	7.33	29.3	7.51	30.0	7.67	30.7
L-Arginine	8.18	32.7	8.36	33.4	7.75	31.0	8.60	34.4	8.81	35.2	9.00	36.0
L-Asparagine-H2O	9.62	38.5	9.83	39.3	9.12	36.5	10.11	40.4	10.36	41.4	10.58	42.3
L-Aspartate	6.97	27.9	7.13	28.5	6.61	26.4	7.33	29.3	7.51	30.0	7.67	30.7
L-Cystine	1.68	6.7	1.72	6.9	1.60	6.4	1.77	7.1	1.81	7.2	1.85	7.4
L-Glutamine	23.57	94.3	24.09	96.4	22.33	89.3	24.77	99.1	25.38	101.5	25.93	103.7
L-Glutamic Acid	28.61	114.4	29.24	117.0	27.11	108.4	30.07	120.3	30.81	123.2	31.48	125.9
Glycine	4.09	16.4	4.18	16.7	3.88	15.5	4.30	17.2	4.40	17.6	4.50	18.0
L-Proline	24.29	97.2	24.82	99.3	23.02	92.1	25.52	102.1	26.16	104.6	26.72	106.9
L-Serine	13.71	54.8	14.01	56.0	12.99	52.0	14.41	57.6	14.77	59.1	15.08	60.3
L-Tyrosine	12.50	50.0	12.78	51.1	11.85	47.4	13.14	52.6	13.47	53.9	13.76	55.0
Corn Starch	315	1260	315	1260	315	1260	315	1260	315	1260	315	1260
Maltodextrin 10	35	140	35	140	35	140	35	140	35	140	35	140
Sucrose	350	1400	350	1400	350	1400	350	1400	350	1400	350	1400
Cellulose	50	0	50	0	50	0	50	0	50	0	50	0
Soybean Oil	25	225	25	225	25	225	25	225	25	225	25	225
Lard	20	180	20	180	20	180	20	180	20	180	20	180
Sodium Bicarbonate	7.5	0	7.5	0	7.5	0	7.5	0	7.5	0	7.5	0
S10026	10	0	10	0	10	0	10	0	10	0	10	0
DiCalcium Phosphate	13	0	13	0	13	0	13	0	13	0	13	0
Calcium Carbonate	5.5	0	5.5	0	5.5	0	5.5	0	5.5	0	5.5	0
Potassium Citrate, 1 H2O	16.5	0	16.5	0	16.5	0	16.5	0	16.5	0	16.5	0
Vitamin Mix V10001	10	40	10	40	10	40	10	40	10	40	10	40
Choline Bitartrate	2	0	2	0	2	0	2	0	2	0	2	0
FD&C Yellow Dye #5	0	0	0.025	0	0	0	0.025	0	0.025	0	0	0
FD&C Red Dye #40	0.05	0	0	0	0.025	0	0.025	0	0	0	0.025	0
FD&C Blue Dye #1	0	0	0.025	0	0.025	0	0	0	0.025	0	0.025	0
Total	1033.29	3940	1033.3	3940	1033.29	3940	1034.01	3943	1033.60	3941	1033.31	3940
Percent EAA (w/w)	19.3		17.5		23.5		15.5		13.2		11.2	
Percent NEAA (w/w)	80.7		82.5		76.5		84.5		86.8		88.8	

Product #	A17041301		A17041302		A17041303	
	Low Lys		Low Thr		Low Trp	
	38.1% EAA, 61.9% NE		39.8% EAA, 60.2% NE		41.2% EAA, 58.8% NE	
	gm	kcal	gm	kcal	gm	kcal
Protein	16.8	17.6	16.8	17.6	16.8	17.6
Carbohydrate	68.7	72.1	68.7	72.1	68.7	72.1
Fat	4.4	10.3	4.4	10.3	4.4	10.3
Total		100.0		100.0		100.0
kcal/gm	3.8		3.8		3.8	
Ingredient (gm)						
L-Histidine-HCl-H2O	4.80	19.2	4.66	18.6	4.56	18.2
L-Isoleucine	7.92	31.7	7.71	30.8	7.54	30.2
L-Leucine	16.59	66.4	16.14	64.6	15.78	63.1
L-Lysine-HCl	3.26	13.0	13.46	53.8	13.16	52.6
L-Methionine	5.35	21.4	5.20	20.8	5.09	20.4
L-Phenylalanine	8.85	35.4	8.61	34.4	8.42	33.7
L-Threonine	7.55	30.2	1.78	7.1	7.18	28.7
L-Tryptophan	2.21	8.8	2.15	8.6	0.62	2.1
L-Valine	9.76	39.0	9.49	38.0	9.28	37.1
L-Alanine	5.35	21.4	5.20	20.8	5.09	20.4
L-Arginine	6.27	25.1	6.10	24.4	5.96	23.8
L-Asparagine-H2O	7.37	29.5	7.17	28.7	7.01	28.0
L-Aspartate	5.35	21.4	5.20	20.8	5.09	20.4
L-Cystine	1.29	5.2	1.26	5.0	1.23	4.9
L-Glutamine	18.07	72.3	17.58	70.3	17.18	68.7
L-Glutamic Acid	21.94	87.8	21.34	85.4	20.87	83.5
Glycine	3.13	12.5	3.04	12.2	2.98	11.9
L-Proline	18.62	74.5	18.11	72.4	17.71	70.8
L-Serine	10.51	42.0	10.23	40.9	10.00	40.0
L-Tyrosine	9.59	38.4	9.33	37.3	9.12	36.5
Corn Starch	315	1260	315	1260	315	1260
Maltodextrin 10	35	140	35	140	35	140
Sucrose	350	1400	350	1400	350	1400
Cellulose	50	0	50	0	50	0
Soybean Oil	25	225	25	225	25	225
Lard	20	180	20	180	20	180
Sodium Bicarbonate	7.5	0	7.5	0	7.5	0
S10026	10	0	10	0	10	0
DiCalcium Phosphate	13	0	13	0	13	0
Calcium Carbonate	5.5	0	5.5	0	5.5	0
Potassium Citrate, 1 H2O	16.5	0	16.5	0	16.5	0
Vitamin Mix V10001	10	40	10	40	10	40
Choline Bitartrate	2	0	2	0	2	0
FD&C Yellow Dye #5	0.025	0	0.025	0	0	0
FD&C Red Dye #40	0.025	0	0	0	0.025	0
FD&C Blue Dye #1	0	0	0.025	0	0.025	0
Total	1033.33	3940	1033.31	3940	1033.32	3940
Percent EAA (w/w)	38.1		39.8		41.2	
Percent NEAA (w/w)	61.9		60.2		58.8	
